# Are EFI data valuable? Evidence from the 2022 FIFA World Cup group stage

**DOI:** 10.5114/biolsport.2024.127382

**Published:** 2023-05-30

**Authors:** Xiaobin Wei, Yifan Zhao, Hui Chen, Peter Krustrup, Morten B Randers, Chong Chen

**Affiliations:** 1 School of Strength and Conditioning Training, Beijing Sport University, Beijing, China; 2 China Institute of Sport Science, Beijing, China; 3 School of sports and health management, Chongqing University of Education, Chongqing, China; 4 Department of Sports Science and Clinical Biomechanics, SDU Sport and Health Sciences Cluster, University of Southern Denmark, Odense, Denmark; 5 Danish Institute for Advanced Study (DIAS), University of Southern Denmark, Odense, Denmark; 6 Sport and Health Sciences, College of Life and Environmental Sciences, University of Exeter, Exeter, United Kingdom; 7 School of Sport Sciences, Faculty of Health Sciences, UiT, The Arctic University of Norway, Tromsø, Norway; 8 Beijing Research Institute of Sport Science, Beijing, China

**Keywords:** Performance Soccer, Match analysis, Football Goal Metrics

## Abstract

The 2022 World Cup used new Enhanced Football Intelligence (EFI) data, stoppage time calculation methods and substitution rules that were introduced by FIFA. The aim of this study is to explore the effectiveness of EFI in match analysis and to identify the key indicators that influence the match and provide a reference for coaches’ tactical design and training. Data were derived from the FIFA website, including EFI data for 48 matches at the group stage of the Qatar World Cup. A total of 46 indicators were used for analysis and the average values of the corresponding indicators for the different competition results were used in the analysis to identify the key index that determines the outcome of the competition. Apart from scoring more goals and having more assists, winning teams had significantly more attempts on target than drawing and losing teams (p < 0.05); Winning teams had significantly more attempts inside the penalty area, completed defensive line breaks and receptions behind the defensive line than losing teams (p < 0.05). There is no difference in possession between matches with different results (p > 0.05). Goals were significantly correlated with completed defensive line breaks and receptions behind the defensive line (r = 0.27–0.30, p < 0.01). Attempts on target was significantly positively correlated with receptions, final third entries and line breaks (r = 0.31–0.67, p < 0.01) and negatively correlated with defensive pressures applied (r = -0.35, p < 0.01). The efficiency of the offense is more important. Teams need to have more receptions, final third entries and line breaks to get more shots on target rather than possession. This study may help coaches to interpret the game from a multi-dimensional perspective and coaches can use EFI to help their teams improve their match performance.

## INTRODUCTION

Football technical indicators reflect a team’s athletic performance and influence the outcome of a match, and therefore receive a great deal of attention from practitioners [1–3]. The FIFA World Cup, as the highest-level soccer tournament at the international level for national teams, has received particular attention from researchers for its influence and competitive nature [1, 4–9].

For example, Castellano et al. [5] combined the 2002, 2006, and 2010 World Cups and found that the number of shots, shots on goal, and possession were the most influential factors in the game, consistent with the results of subsequent analyses of 2014 and 2018 [1, 4]. Liu et al. [1] found in 2014 World Cup that possession and short passing increased winning possibilities by 11% and 24%, respectively, while crosses and dribbles decreased the probability of winning by 29% and 12%, respectively. Yi et al. [10] found accordingly a higher probability of winning for teams with a possession style in the 2018 World Cup. An analysis of possession by high level teams at the 2010–2018 World Cup found that possession in one’s own defensive zone increases the likelihood of goals through multiple passes in short periods of time [9].

However, there is some dispute in the research about the simple use of higher or lower possession. In some tournaments, superior possession does not lead to higher win rates [11], and distinguishing the main areas when in possession is more critical [12]. In addition, recent studies found that crosses have a positive impact on the analysed in this outcome of matches [13, 14], contrary to previous findings [1, 15]. More precisely, the 2018 World Cup data show a higher probability of scoring from out-swinging crosses and the losing teams prefer to take middle crosses and late crosses [16]. Moreover, 69.9% of goals were scored from short passes, 13.6% from long passes and 16.5% from mixed passes [17].

To sum up, the notational analysis of the World Cup has produced some results, but the indicators are relatively conventional. Conclusions such as more shots on target help teams win are difficult for teams to apply in practice. There is a need to find more nuanced technical indicators that can help teams improve their performance.

Based on this, FIFA has launched Enhanced Football Intelligence (EFI) to provide a more intelligent and refined reference for match analysis. In the 2022 World Cup, FIFA assembled a data analysis team and started to use this indicator for relevant statistics. Each game will have its unique analytical data during and after the live broadcast. As new data to be used from 2022, the EFI has different characteristics from the previous data. For example, defensive height can distinguish a team’s defensive starting position; possession in contest adds an insightful third dimension to possession statistics, which cannot be clearly calculated at the moment of scramble in previous data. EFI has several times more data points in the competition than previous data counting methods. The factual data in the match can be processed in 2 seconds with its high-speed algorithm and fed back to the officials quickly. It can provide both factual data during the match and during the post-match evaluation. If EFI data are combined with video and other metrics such as running patterns, it will provide practitioners with a clearer understanding of the game. In fact, EFI is already being used in the Women’s U20 World Cup in August 2022, providing a powerful aid for match analysts.

Considering that EFI has just been used in the competition, it is necessary to study its effectiveness in analysing the match. Therefore, the aim of this study is to compare the differences in technical indicators between the results of different matches and the relationship between EFI and attempts on target.

## MATERIALS AND METHODS

### Sample and variable

The data are taken from the official FIFA website (https://www.fifa.com/fifaplus/en/tournaments/mens/worldcup/qatar2022), which is freely available. Another four success rate figures (line breaks, defensive line breaks, passes and crosses) were calculated from FIFA data for analysis. During matches in the knockout rounds more goals were scored than during matches group stage (3.1 vs 2.5 goals). Since almost one third of the knockout games went into extra time, and the data provided by FIFA are inclusive of extra time data and not for the 90 minutes separate, only matches in the group stage (48 games, 96 cases (38 won, 20 drew, 38 lost)) were selected as the object of study. The definition of the EFI data is provided on the official FIFA website (https://www.fifatrainingcentre.com/en/fwc2022/efi-metrics/efi-metrics-pdfs.php) and Table 1 shows the variables analysed in this paper. Since this study is an observational study without any intervention on the subjects, no ethical proof is required.

**TABLE 1:** Selected technical match variables.

Variable		Measurements
Goal
	Goal (n)	A team succeeds in scoring a goal
	Conceded (n)	Goal scored by opponent
	Goal Inside the Penalty Area (n)	The shot before the goal took place in the penalty area
	Goal Outside the Penalty Area (n)	The shot before the goal took place outside the penalty area
	Assists (n)	A pass that gets converted into a goal by another player

Attempts	Attempts (n)	An attempt to score a goal
	Attempts On Target (n)	Shots where the target is within the range of the goal
	Attempts Off Target (n)	Shots where the target is outside the range of the goal
	Attempts Inside the Penalty Area (n)	Shots that occur inside the penalty area
	Attempts Outside the Penalty Area (n)	Shots that occur outside the penalty area

Possession	Total (%)	Percentage of total time that a team has been in full control of the ball
	In Contest (%)	The ball was not always fully controlled by both sides of the game and the percentage of the total game time that was spent with contested possessions.

Final Third Entries	Left Channel (n)	The ball is successfully distributed or carried into the last third of the left channel of the final third, which consists of the left sideline, the extension of the left penalty area line and the offensive third of the field.
	Left Inside Channel (n)	The ball is successfully distributed or carried into the last third of the left inside channel of the final third, which consists of the extended area of the left goal area line, the extended area of the left penalty area line and the offensive third of the pitch.
	Central Channel (n)	The ball is successfully distributed or carried into the last third of the central channel of the final third, which consists of the extended area of the left goal area line, the extended area of the right goal area line, and the offensive third of the pitch.
	Right Inside Channel (n)	The ball is successfully distributed or carried into the last third of the right inside channel of the final third, which consists of the extended area of the right goal area line, the extended area of the right penalty area line and the offensive third of the pitch.
	Right Channel (n)	The ball is successfully distributed or carried into the last third of the right channel of the final third, which consists of the right sideline, the extension of the right penalty area and the offensive third of the field.

Offers to Receive	Total (n)	A clear and deliberate action performed in an attempt to receive the ball
	Offers to Receive In Behind (n)	A clear and deliberate action performed in an attempt to receive the ball behind the defensive line of the opponent
	Offers to Receive In Between (n)	A clear and deliberate action performed in an attempt to receive the ball between the first line and the defensive line of the opponent
	Offers to Receive In Front (n)	A clear and deliberate action performed in an attempt to receive the ball in front of the first line of the opponent

Receptions	Receptions Between Midfield and Defensive Lines (n)	The ball has been received between the opponents’ midfield and defensive line
	Receptions Behind the Defensive Line (n)	The ball has been received behind the opponents’ defensive line

Line Breaks	Attempted Line Breaks (n)	The team attempts to pass/cross or carry the ball past the last player in one of the lines of the defending team
	Completed Line Breaks (n)	The team successfully passes/crosses or carries the ball past the last player in one of the lines of the defending team
	Success Rate of Line Breaks (%)	Successful line breaks as a percentage of attempted line breaks
	Attempted Defensive Line Breaks (n)	The team attempts to pass/cross or carry the ball past the last player in the defensive line of the defending team
	Completed Defensive Line Breaks (n)	The team successfully passes/crosses or carries the ball past the last player in the defensive line of the defending team
	Success Rate of Defensive Line Breaks (%)	Successful defensive line breaks as a percentage of attempted defensive line breaks

Fouls	Yellow Cards (n)	Player shown yellow card by referee for foul and other offences
	Red Cards (n)	Player shown red card by referee for foul and other offences
	Fouls Against (n)	Any infringement that is penalised as foul play by a referee
	Offsides (n)	Appeared in an offside position, which was called by the referee

Pass/Cross	Passes (n)	Short passes aimed at teammates
	Passes Completed (n)	Successful pass to a teammate
	Success Rate of Passes (%)	Successful passes as a proportion of total passes
	Crosses (n)	Long passes aimed at teammates
	Crosses Completed (n)	Successful crosses to a teammate
	Success Rate of Crosses (%)	Successful crosses as a proportion of total crosses

Others index	Switches of Play Completed (n)	Successfully completed the attack through switches
	Corners (n)	The ball passes over the goal line, on the ground or in the air, having last touched a player of the defending team, and a goal is not scored
	Free Kicks (n)	The ball is given to a member of one side to kick because a member of the other side has broken a rule
	Penalties Scored (n)	Goal scored by penalties
	Goal Preventions (n)	Actions a goalkeeper takes when attempting to prevent the concession of a goal
	Own Goal (n)	A goal scored by the defending team
	Forced Turnovers (n)	The attacking team loses position of the ball due to pressure being applied by the defending team
	Defensive Pressures Applied (n)	Defensive pressure applied towards an attacker in possession of the ball

### Statistical analysis

Data were processed using SPSS 26.0 (IBM, Armonk, NY, USA), and data were expressed as mean ± standard deviation. Normality was checked by Kolmogorov-Smirnov tests. To compare the difference between win, draw and loss figures, one-way ANOVA was used for normal data, the Levene test was used for the homogeneity-of-variance test, the least significant difference (LSD) test was used for post-hoc tests when the variance was homoscedastic, and Tamhane’s T2 test was used when the variance was not homoscedastic. The chi-square test was used to determine the difference between teams with different possession rates, passing success rates, and line breaking success rate. K-sample independent tests were used for non-normal data. A non-parametric correlation test was performed using Spearman correlation. The criteria for correlation are as follows: r = 0.1–0.29 = small, 0.3–0.49 = medium, 0.5–0.69 = large, 0.7–0.89 = very large, 0.9–0.99 = almost perfect, and 1 = perfect [18]. The significance level was defined as *p* < 0.05.

## RESULTS

In the group stage, there was a total of 120 goals, of which 36% were scored in the first half. Among all the indexes provided by FIFA, only goals, goals conceded, goals inside the penalty area, goals outside the penalty area, assists and attempts on target were significantly different between winning, drawing and losing teams (p < 0.05). Winning teams had significantly more goals, goals inside the penalty area, assists, and attempts on target than drawing and losing teams (p < 0.05). Winning teams scored significantly more goals outside the penalty area, had more attempts inside the penalty area, more receptions behind the defensive line, completed more defensive line breaks, had a higher success rate of defensive line breaks, and more forced turnovers than losing teams (p < 0.05). Drawing teams had significant fewer yellow cards than losing teams (p < 0.05). No significant difference was found in other data (Tables 2 and 3).

**TABLE 2:** FIFA EFI metrics for winning, drawing and losing teams (mean ± SD)

Index	Win	Draw	Lose	F	K	p
Goal	2.2 ± 1.4*^	0.6 ± 0.9	0.6 ± 0.8		40.767	0.000#
Conceded	0.6 ± 0.8*	0.6 ± 0.9&	2.2 ± 1.4		40.767	0.000#
Goal Inside the Penalty Area	2.0 ± 1.4*^	0.5 ± 0.8	0.5 ± 0.7		36.953	0.000#
Goal Outside the Penalty Area	0.2 ± 0.4*	0.1 ± 0.3	0.0 ± 0.2		7.762	0.021#
Assists	1.6 ± 1.3*^	0.5 ± 1.0	0.4 ± 0.4		31.326	0.000#
Attempts	12.2 ± 6.6	9.6 ± 3.5	10.3 ± 5.7		2.075	0.354
Attempts On Target	5.0 ± 2.9*^	2.1 ± 2.1	3.1 ± 2.1		10.781	0.005#
Attempts Off Target	4.8 ± 3.1	4.4 ± 2.1	5.0 ± 2.9		0.310	0.856
Attempts Inside the Penalty Area	7.8 ± 4.7*	6.2 ± 3.1	5.9 ± 4.2		4.548	0.103
Attempts Outside the Penalty Area	4.4 ± 3.6	3.4 ± 1.3	4.5 ± 2.7		2.194	0.334
Final Third Entries						
Left Channel	13.5 ± 7.8	12.3 ± 5.3	13.4 ± 7.0		0.037	0.982
Left Inside Channel	4.7 ± 3.2	3.7 ± 2.2	4.8 ± 2.8		2.006	0.367
Central Channel	5.0 ± 3.1	4.7 ± 3.3	4.5 ± 2.6		0.375	0.829
Right Inside Channel	5.0 ± 3.5	3.8 ± 1.6	4.3 ± 2.4		0.908	0.635
Right Channel	12.2 ± 5.3	11.4 ± 5.4	11.4 ± 6.8	0.224		0.800
Offers to Receive	568.5 ± 204.2	554.9 ± 105.1	553.5 ± 177.7	0.078		0.925
Offers to Receive In Behind	126.9 ± 40.7	116.0 ± 23.2	121.5 ± 39.5	0.579		0.562
Offers to Receive In Between	218.1 ± 76.2	225.6 ± 63.1	213.5 ± 59.7	0.210		0.811
Offers to Receive In Front	223.6 ± 103.7	213.3 ± 51.6	218.5 ± 109.6		0.132	0.936
Receptions Between Midfield and Defensive Lines	99.0 ± 30.8	92.5 ± 20.5	94.1 ± 29.1	0.451		0.638
Receptions Behind the Defensive Line	12.6 ± 7.0*	10.1 ± 4.8	9.4 ± 6.1		5.560	0.062
Attempted Line Breaks	167.2 ± 32.6	172.5 ± 20.5	164.6 ± 34.9	0.404		0.669
Completed Line Breaks	111.9 ± 34.6	109.4 ± 26.4	104.3 ± 32.1		0.514	0.773
Attempted Defensive Line Breaks	18.9 ± 7.0	19.1 ± 5.5	16.9 ± 7.0	1.109		0.334
Completed Defensive Line Breaks	10.8 ± 5.6*	9.7 ± 4.6	8.6 ± 6.0		4.585	0.101
Yellow Cards	1.6 ± 1.5	1.3 ± 1.1&	2.1 ± 1.6		4.344	0.114
Red Cards	0.1 ± 0.2	0	0			
Fouls Against	11.8 ± 3.7	11.6 ± 3.1	12.6 ± 5.0	0.550		0.579
Offsides	1.8 ± 1.4	1.5 ± 1.6	2.2 ± 2.1		1.688	0.430
Passes	484.8 ± 192.7	485.5 ± 104.9	484.4 ± 159.0		0.467	0.792
Passes Completed	419.8 ± 192.3	411.8 ± 108.4	411.0 ± 159.1		0.225	0.893
Crosses	18.1 ± 7.9	18.3 ± 7.2	18.2 ± 8.3		0.014	0.993
Crosses Completed	4.7 ± 3.3	3.9 ± 2.6	4.5 ± 3.0		1.089	0.580
Switches of Play Completed	6.3 ± 3.3	6.5 ± 4.2	5.9 ± 4.0		0.635	0.728
Corners	4.9 ± 3.1	4.6 ± 2.5	4.1 ± 3.0		2.047	0.359
Free Kicks	14.1 ± 5.1	13.0 ± 3.2	13.1 ± 4.7		1.411	0.494
Penalties Scored	0.1 ± 0.3	0.1 ± 0.2	0.1 ± 0.3		1.176	0.555
Goal Preventions	10.6 ± 5.8	9.9 ± 3.7	12.5 ± 6.6		1.890	0.389
Own Goal	0.1 ± 0.2	0	0			
Forced Turnovers	72.3 ± 11.1	71.0 ± 13.6	65.9 ± 13.5		4.087	0.130
Defensive Pressures Applied	288.1 ± 92.4	280.7 ± 55.5	282.2 ± 87.7	0.069		0.933

* : Significant difference between win and lose;

^ : Significant difference between win and draw;

& :Significant difference between draw and lose.

# : significant difference between groups

**TABLE 3:** Chi-square test of different match results

Index	Win	Draw	lose	χ^2^	p
Possession (%)	43.8 ± 13.9	43.1 ± 9.8	44.3 ± 13.3	84.653	0.460
Success Rate of Line Breaks (%)	66.0 ± 10.2	62.8 ± 9.4	62.5 ± 9.1	183.579	0.453
Success Rate of Defensive Line Breaks (%)	55.2 ± 15.4	48.7 ± 13.1	46.4 ± 20.8	107.865	0.816
Success Rate of Passes (%)	85.0 ± 8.7	83.9 ± 4.9	83.3 ± 6.1	192.000	0.446
Success Rate of Crosses (%)	26.1 ± 13.4	20.1 ± 10.4	23.9 ± 9.6	98.311	0.639

**FIG. 1:**
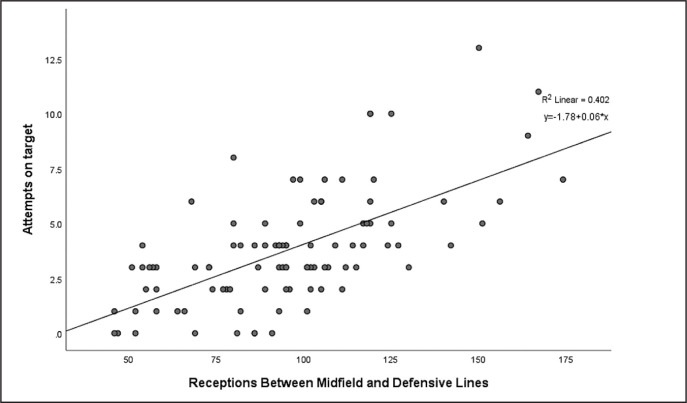
Regression analysis of Attempts and Receptions Between Midfield and Defensive Lines

**FIG. 2:**
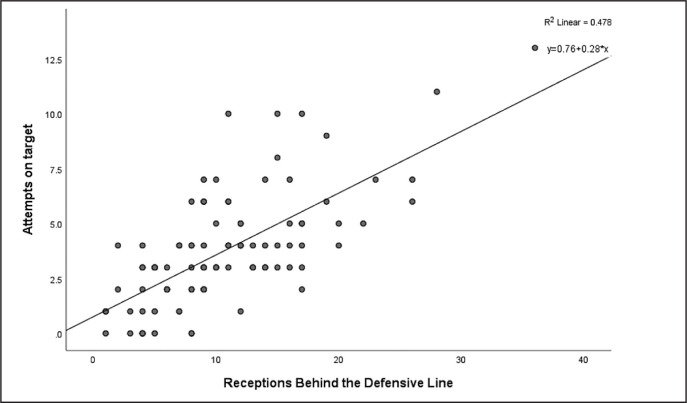
Regression Analysis of Attempts and Receptions Behind the Defensive Line

The number of goals was correlated with completed defensive line breaks and receptions behind the defensive line (p < 0.01) with no other EFI data correlated with number of goals (Table 4). Attempts on target were significantly positively correlated with attempted line breaks, completed line breaks, attempted defensive line breaks, completed defensive line breaks, receptions between midfield and defensive lines, receptions behind the defensive line and all the area of final third entries (r = 0.31–0.67, p < 0.01), and negatively correlate with defensive pressures applied (r = -0.35, p < 0.01) (Table 4). A two-factor linear regression model was constructed on the attempts on target and receiver data, as these indicators are particularly important for technical and tactical purposes and have the highest correlation coefficient (Figures 1 and 2). The R^2^ for the attempts on target was 0.40 for receptions between midfield and defensive lines and 0.48 for receptions behind the defensive line.

**TABLE 4:** Correlation Between EFI Indicator and Goals/Attempts On Target

	Line Breaks				Receptions		Final Third Entries					FT	DPA

	ALB	CLB	ADLB	CDLB	BMDL	BDL	LC	LIC	CC	RIC	RC		
Goals	0.037	0.104	0.137	0.265**	0.191	0.298**	-0.041	0.097	0.064	0.083	0.021	0.161	-0.054
AOT	0.417**	0.486**	0.480**	0.605**	0.621**	0.667**	0.373**	0.466**	0.395**	0.335**	0.308**	-0.036	-0.347**

** represents P < 0.01. AOT = Attempts On Target, ALB = Attempted Line Breaks, CLB = Completed Line Breaks, ADLB = Attempted Defensive Line Breaks, CDLB = Completed Defensive Line Breaks, BMDL = Between Midfield and Defensive Lines, BDL = Behind the Defensive Line, LC = Left Channel, LIC = Left Inside Channel, CC = Central Channel, RIC = Right Inside Channel, RC = Right Channel, FT = Forced Turnovers, DPA = Defensive Pressures Applied.

## DISCUSSION

This study is the first to examine the validity of EFI for analysing matches, and this study compares the differences in technical indicators for different match outcomes at the 2022 World Cup group stage.

We found that most goals and attempts related indicators can distinguish the outcome of a match, in line with previous studies [1, 5, 6]. However, there is no difference in the total number of attempts between winning, drawing and losing, in contrast to previous studies in both the male and female FIFA World Cup [1, 5, 19]. This suggests that the efficiency of attempts was even more important in determining the outcome of the game. Also, no significant difference (p > 0.05) in the number of corners and free kicks was found between winning, drawing and losing, in contrast to the study of the LaLiga [20] and

Women’s World Cup [19]. This may be due to increasing prevalence of intensive defending making set pieces become more and more crucial, and successful teams are more efficient at scoring from set pieces than their less successful opponents [21]. In addition, drawing teams but not winning teams had significantly fewer yellow cards compared to losing teams, which is partially different from previous studies [6, 22]. The main reason may be that most of the draws in the 2022 World Cup occurred in the last match of the group. With the group’s advancing form clear, both sides deliberately adopted a low-aggressive strategy to prevent players from being injured and banned from the next stage, thus resulting in a significant drop in the number of yellow cards. For the possession after the new calculation method based on EFI, no differences were found between the different results of the match (p > 0.05), in contrast to previous studies [4, 5, 10]. The reason for this is, on the one hand, that the possession rates of both sides are closer after the inclusion of the “in contest” moments. On the other hand, it is difficult to convert pos-session into an offensive advantage with the strategy of counterattack being more prevalent due to the increased intensity of the game and the density of player coverage [23]. For intensive and high density defence, it will not only put high pressure on the possession team but also increase the success rate of counterattack of the opponents [24]. Thus, many teams will voluntarily give up possession, especially against strong teams [25]. The number of passes and success rate of passes are considered to be among the most important signs of ball control [7, 26], but the indicator was not found to be associated with differences between the results of different matches, further supporting the conclusion that possession plays a limited role.

In addition, we found that receptions behind the defensive line and completed defensive line breaks made a difference between winning and losing teams (p < 0.05). Higher rates of these mean that the team has more chances to get into the penalty area and attempts, and therefore will have more chances to score and win the game. Although other EFI data did not account for differences in results across matches, this does not mean that other EFI data are not meaningful, as match performance and results are also influenced by other factors such as opponents and judgements of referees [27–29]. We found that almost all post-match EFI indicators are correlated with attempts on target (p < 0.01), except for forced turnovers. However, the difference between forced turnovers in winning and losing teams approached the significance level (p = 0.05), consistent with the findings in LaLiga teams [20]. The reasons for this are manifold; marker, location, individual errors, pressure, conditioning [30], etc. all affect the forced turnover result of teams, especially in a knock-out tournament like the World Cup where matches are played at short intervals. Moreover, as the Qatar World Cup is held in December, the league became more congested before the World Cup 2022 than previous World Cups, which increased the physical burden of the players [31, 32]. Therefore, fewer teams may be adopting an aggressive high-pressure strategy, leading to forced turnovers in the group stage being not too decisive.

Finally, line break, reception in the key area and final third entry all have a medium to large positive correlation with attempts on target, indicating that performing these actions as much as possible will help the team get more attempts on target to a greater extent. Studies of high-level players have shown that penetrating performances and exploiting gaps in the defensive line can increase a team’s chances of scoring goals [33, 34], supporting our results. In fact, the results show that receptions between midfield and defensive lines and behind the defensive line explain 40% and 47% of the variance in shots on target, respectively. A relatively high figure considering that there are other events after the reception that can affect the likelihood of a shot. Additionally, more entries into final third area mean that the team has more chances to threaten the goal and therefore correlates moderately with the number of attempts on target. This may also be the reason why the percentage of possession in this area was found to be more critical compared to total possession [12].

In general, this study provides evidence for practitioners to improve their match performance. Possession does not determine the outcome of a game, and teams need to be efficient in attack and give up possession appropriately. At the same time, the efficiency of the shot is more important than the number of shots. This study also indicates that the coach needs to improve the tactics of players in the game, opting to receive the ball between the lines whenever possible, to enhance the team’s shooting opportunities. Meanwhile, this study also provides ideas for athlete development, i.e., developing more awareness and ability to catch the ball at the line of defence in training.

One of the limitations of this study is that it did not incorporate physical fitness data. However, technical indicators are more likely to predict a team’s success than physical indicators [35, 36], and thus this study can still provide an important reference for practitioners. The inability to compare the group stage with the knockout stage is another limitation of this study. Future research can integrate technical indicators with physical and other contextual information such as opponent level, weather, and altitude. It is also possible to use video analysis to find out which technical indicators at specific moments of the game can help the team gain a greater advantage. This will provide more detailed guidance on team tactical options.

## CONCLUSIONS

There were significant differences in goal-related variables between match outcomes. In addition, the winning team had more defensive line breaks and receptions at the group stage of the 2022 World Cup. Receptions, final third entries and line breaks have a medium to high correlation with attempts on target and can provide important information for practitioners. Coaches need to identify the key indicators that affect the outcome of a match, rather than focusing on indicators such as possession that do not give a clear advantage. To conclude, the EFI provides a new reference for match analysis, which practitioners can use to better improve their team’s match performance.

## References

[1] Liu H, Gomez MÁ, Lago-Peñas C, Sampaio J. Match statistics related to winning in the group stage of 2014 Brazil FIFA World Cup. J Sports Sci. 2015; 33(12):1205–13. doi: 10.1080/02640414.2015.1022578. 25793661

[2] Sarmento H, Figueiredo A, Lago-Peñas C, Milanovic Z, Barbosa A, Tadeu P, et al. Influence of Tactical and Situational Variables on Offensive Sequences During Elite Football Matches. J Strength Cond Res. 2018; 32(8):2331–9. doi: 10.1519/jsc.0000000000002147. 28737587

[3] Errekagorri I, Castellano J, Echeazarra I, López-Del Campo R, Resta R. A longitudinal analysis of technical-tactical and physical performance of the teams in the Spanish LaLiga Santander: An eight-season study. Biol Sport. 2022; 39(2):389–96. Epub 2022/03/22. doi: 10.5114/biolsport.2022.105331. 35309534 PMC8919887

[4] Alves DL, Osiecki R, Palumbo DP, Moiano-Junior JVM, Oneda G, Cruz R. What variables can differentiate winning and losing teams in the group and final stages of the 2018 FIFA World Cup? Int J Perform Anal Sport. 2019; 19(2):248–57. doi: 10.1080/24748668.2019.1593096.

[5] Castellano J, Casamichana D, Lago C. The Use of Match Statistics that Discriminate Between Successful and Unsuccessful Soccer Teams. J Hum Kinet. 2012; 31:139–47. doi: 10.2478/v10078-012-0015-7. 23487020 PMC3588662

[6] Rumpf MC, Silva JR, Hertzog M, Farooq A, Nassis G. Technical and physical analysis of the 2014 FIFA World Cup Brazil: winners vs. losers. J Sports Med Phys Fitness. 2017; 57(10):1338–43. doi: 10.23736/S0022-4707.16.06440-9. 27167712

[7] da Mota GR, Thiengo CR, Gimenes SV, Bradley PS. The effects of ball possession status on physical and technical indicators during the 2014 FIFA World Cup Finals. J Sports Sci. 2016; 34(6):493–500. doi: 10.1080/02640414.2015.1114660. 26703781

[8] Vergonis A, Balasas D, Michailidis Y, Metaxas T. The significant role of scoring from set plays in the 2018 FIFA World Cup. J Sports Med Phys Fitness. 2021; 61(11):1448–53. doi: 10.23736/s0022-4707.20.11788-2. 33969954

[9] Taha T, Ali AY. Greater numbers of passes and shorter possession durations result in increased likelihood of goals in 2010 to 2018 World Cup Champions. PLoS One. 2023; 18(1):e0280030. doi: 10.1371/journal.pone.0280030. 36607978 PMC9821415

[10] Yi Q, Gómez MA, Wang L, Huang G, Zhang H, Liu H. Technical and physical match performance of teams in the 2018 FIFA World Cup: Effects of two different playing styles. J Sports Sci. 2019; 37(22):2569–77. doi: 10.1080/02640414.2019.1648120. 31354060

[11] Collet C. The possession game? A comparative analysis of ball retention and team success in European and international football, 2007–2010. J Sports Sci. 2013; 31(2):123–36. doi: 10.1080/02640414.2012.727455. 23067001

[12] Casal CA, Anguera MT, Maneiro R, Losada JL. Possession in Football: More Than a Quantitative Aspect – A Mixed Method Study. Front Psychol. 2019; 10:501. doi: 10.3389/fpsyg.2019.00501. 30936844 PMC6431675

[13] Bai L, Gedik R, Egilmez G. What does it take to win or lose a soccer game? A machine learning approach to understand the impact of game and team statistics. J Oper Res Soc. 2022:1–22. doi: 10.1080/01605682.2022.2110001.

[14] Lepschy H, Woll A, Wäsche H. Success Factors in the FIFA 2018 World Cup in Russia and FIFA 2014 World Cup in Brazil. Front Psychol. 2021; 12:638690. doi: 10.3389/fpsyg.2021.638690. 33767649 PMC7985168

[15] Lago-Peñas C, Lago-Ballesteros J, Dellal A, Gómez M. Game-related statistics that discriminated winning, drawing and losing teams from the Spanish soccer league. J Sports Sci Med. 2010; 9(2):288. 24149698 PMC3761743

[16] Mitrotasios M, Kubayi A, Armatas V, Larkin P. Analysis of Crossing Opportunities at the 2018 FIFA World Cup. Montenegrin J Sports Sci Med. 2022; 11(1):43–52. doi: 10.26773/mjssm.220305.

[17] Kubayi A. Analysis of Goal Scoring Patterns in the 2018 FIFA World Cup. J Hum Kinet. 2020; 71:205–10. doi: 10.2478/hukin-2019-0084. 32148584 PMC7052713

[18] Hopkins W, Marshall S, Batterham A, Hanin J. Progressive statistics for studies in sports medicine and exercise science. Med Sci Sport Exer. 2009; 41(1):3. doi: 10.1249/MSS.0b013e31818cb278. 19092709

[19] Kubayi A, Larkin P. Technical performance of soccer teams according to match outcome at the 2019 FIFA Women’s World Cup. Int J Perform Anal Sport. 2020; 20(5):908–16. doi: 10.1080/24748668.2020.1809320.

[20] Brito de Souza D, López-Del Campo R, Blanco-Pita H, Resta R, Del Coso J. An extensive comparative analysis of successful and unsuccessful football teams in LaLiga. Front Psychol. 2019; 10:2566. doi: 10.3389/fpsyg.2019.02566. 31781011 PMC6856952

[21] Pulling C, Robins M, Rixon T. Defending corner kicks: analysis from the English Premier League. Int J Perform Anal Sport. 2013; 13(1):135–48. doi: 10.1080/24748668.2013.11868637.

[22] Lago-Peñas C, Lago-Ballesteros J, Rey E. Differences in performance indicators between winning and losing teams in the UEFA Champions League. J Hum Kinet. 2011; 27(1):135–46. doi: 10.2478/v10078-011-0011-3

[23] Wallace JL, Norton KI. Evolution of World Cup soccer final games 1966–2010: Game structure, speed and play patterns. J Sci Med Sport. 2014; 17(2):223–8. doi: 10.1016/j.jsams.2013.03.016. 23643671

[24] Gómez MA, Gómez-Lopez M, Lago C, Sampaio J. Effects of game location and final outcome on game-related statistics in each zone of the pitch in professional football. Eur J Sport Sci. 2012; 12(5):393–8. doi: 10.1080/17461391.2011.566373.

[25] Wang SH, Qin Y, Jia Y, Igor KE. A systematic review about the performance indicators related to ball possession. PLoS One. 2022; 17(3):e0265540. doi: 10.1371/journal.pone.0265540. 35298562 PMC8929629

[26] Bradley PS, Lago-Peñas C, Rey E, Gomez Diaz A. The effect of high and low percentage ball possession on physical and technical profiles in English FA Premier League soccer matches. J Sports Sci. 2013; 31(12):1261–70. doi: 10.1080/02640414.2013.786185. 23697463

[27] Lago-Peñas C, Gómez-López M. The influence of referee bias on extra time in elite soccer matches. Percept Mot Skills. 2016; 122(2):666–77. doi: 10.1177/0031512516633342. 27166341

[28] Aquino R, Martins GHM, Vieira LHP, Menezes RP. Influence of match location, quality of opponents, and match status on movement patterns in Brazilian professional football players. J Strength Cond Res. 2017; 31(8):2155–61. doi: 10.1519/JSC.0000000000001674. 28737610

[29] Taylor JB, Mellalieu SD, James N, Shearer DA. The influence of match location, quality of opposition, and match status on technical performance in professional association football. J Sports Sci. 2008; 26(9):885–95. doi: 10.1080/02640410701836887. 18569554

[30] Bortnik L, Burger J, Rhodes D. The mean and peak physical demands during transitional play and high pressure activities in elite football. Biol Sport. 2022; 40(1):1055–64. doi: 10.5114/biolsport.2023.112968. PMC9536371 36247966

[31] Arruda AF, Carling C, Zanetti V, Aoki MS, Coutts AJ. Effects of a very congested match schedule on body-load impacts, accelerations, and running measures in youth soccer players. Int J Sport Physiol. 2015; 10(2):248–52. doi: 10.1123/ijspp.2014-0148. 25117378

[32] Carling C, Gregson W, McCall A, Moreira A, Wong DP, Bradley PS. Match running performance during fixture congestion in elite soccer: research issues and future directions. Sports Med. 2015; 45(5):605–13. doi: 10.1007/s40279-015-0313-z. 25694027

[33] González-Rodenas J, Aranda-Malavés R, Tudela-Desantes A, Calabuig Moreno F, Casal CA, Aranda R. Effect of Match Location, Team Ranking, Match Status and Tactical Dimensions on the Offensive Performance in Spanish ‘La Liga’ Soccer Matches. Front Psychol. 2019; 10:2089. doi: 10.3389/fpsyg.2019.02089. 31572270 PMC6751314

[34] Tenga A, Mortensholm A, O’Donoghue P. Opposition interaction in creating penetration during match play in elite soccer: evidence from UEFA champions league matches. Int J Perform Anal Sport. 2017; 17(5):802–12. doi: 10.1080/24748668.2017.1399326.

[35] Bradley PS, Carling C, Diaz AG, Hood P, Barnes C, Ade J, et al. Match performance and physical capacity of players in the top three competitive standards of English professional soccer. Hum Mov Sci. 2013; 32(4):808–21. doi: 10.1016/j.humov.2013.06.002. 23978417

[36] Carling C. Interpreting physical performance in professional soccer match-play: should we be more pragmatic in our approach? Sports Med. 2013; 43(8):655–63. doi: 10.1007/s40279-013-0055-8. 23661303

